# Where functional MRI stops, metabolism starts

**DOI:** 10.7554/eLife.78327

**Published:** 2022-04-25

**Authors:** Polytimi Frangou, William T Clarke

**Affiliations:** 1 Wellcome Centre for Integrative Neuroimaging, University of Oxford Oxford United Kingdom

**Keywords:** perception, human brain metabolism, lactate, BOLD fMRI, magnetic resonance spectroscopy, Human

## Abstract

Combining techniques that track blood oxygenation and biochemicals during neuronal activity reveals how the brain computes perceived and unperceived stimuli.

**Related research article** DiNuzzo M, Mangia S, Moraschi M, Mascali D, Hagberg GE, Giove F. 2022. Perception is associated with the brain’s metabolic response to sensory stimulation. *eLife* **11**:e71016. doi: 10.7554/eLife.71016.

Look around you, the room you are in, the people and objects that surround you. As you do, the visual cortex at the back of your brain becomes active and starts to process these perceived stimuli in multiple stages of increased complexity: lines and colours are computed by the primary visual regions which then forward this information to secondary areas that piece together these simple features into people and objects.

Visual information is usually presented at a rate that your visual cortex can process, but this is not always the case. The very monitor you are reading this article on, for example, probably updates its display at least 60 times a second. Such a fast rate cannot be perceived, which is why we only register a steady image or a smooth video rather than continuous flickering. Unperceived stimulation is not fully processed by the visual stream, but it may still elicit activity in the primary visual cortex. In fact, little is known about how the brain handles unperceived signals; this is partly because current approaches in brain imaging cannot distinguish between perceived and unperceived signals in the visual cortex.

Now, in eLife, Federico Giove and colleagues based in Italy, Germany and the United States – including Mauro DiNuzzo and Silvia Mangia as joint first authors – report having leveraged two magnetic resonance imaging techniques to explore how to best track the way energy is supplied to the visual cortex for perception (DiNuzzo et al., 2022).

Brain areas that process information need glucose and oxygen to replenish the energy they consume, with these substances being provided by blood vessels coupled to active neurons. Tracking this neurovascular response using functional magnetic resonance imaging (fMRI) and blood-oxygen-level dependent (BOLD) imaging allows scientists to detect active brain regions non-invasively. However, these approaches can only produce a surrogate signal that reflects the brain’s change in oxygen demands; they do not describe neuronal activity per se. In fact, measured changes in BOLD-fMRI are non-specific: they may reflect energy consumption in excitatory neurons, in inhibitory interneurons, or even in a combination of the two (Logothetis, 2008; Moon et al., 2021). For instance, visual tasks with similar neurovascular responses can actually encompass different local inhibitory processes (Frangou et al., 2018). BOLD-fMRI alone may therefore not be sufficient to describe complex cognitive processes at the neuronal level, and to reveal how the same brain regions compute perceived and unperceived stimuli differently.

Examining the biochemical clues left by neural activity could help to overcome this limitation. The levels of lactate and glutamate – two molecules involved in neural processes – can be used to directly assess how metabolic systems change in active areas of the brain. This can be measured using functional magnetic resonance spectroscopy (fMRS), a technique which quantifies chemical compounds within a three-dimensional volume. As recent studies show, combining BOLD-fMRI with fMRS that records glutamate and lactate concentrations can help to dissect the neural processes at play in active brain areas (Mangia et al., 2007; Schaller et al., 2014; Ip et al., 2017; Boillat et al., 2020; Koush et al., 2021).

DiNuzzo et al. capitalized on these methodological advances and harnessed BOLD-fMRI combined with fMRS to examine the brains of participants watching green/red checkerboards that switched their colours either slowly (7.5 Hertz; perceived stimuli) or quickly (30 Hertz; unperceived stimuli). Both perceived and unperceived stimuli were carefully designed to elicit similar neurovascular responses in the primary visual cortex. And, indeed, the BOLD-fMRI signal in the primary visual cortex was the same between perceived and unperceived stimuli (Figure 1a), both within the anatomically defined primary visual cortex and in the three-dimensional volume used in fMRS. Finer measures of the neurovascular response, such as when it started or when it peaked, also did not differ between perceived and unperceived stimulations. Measuring BOLD-fMRI signal in secondary visual areas corroborated the notion that unperceived stimuli are not processed further in the visual stream (Figure 1c). This suggests that the neurovascular response in the primary visual cortex cannot, by itself, distinguish between different local processing of perceived and unperceived stimuli.

**Figure 1.**
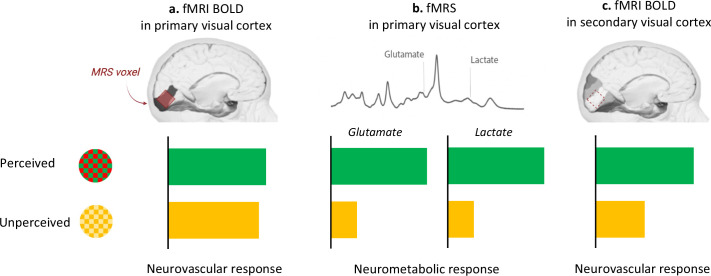
Perception drives the brain’s neurometabolic but not neurovascular response to visual stimulation. Participants were presented with checkerboards that flickered between different colours. In the perceived condition, the red and green checkerboards switched colours slowly (7.5 Hertz); in the unperceived condition, they switched quickly (30 Hertz) and appeared as static yellow. (**a**) The neurovascular response (as measured by BOLD-fMRI) was not different between the two conditions (green bar, perceived; yellow bar, unperceived) in the primary visual cortex, defined as an anatomical brain region (dark grey) or as the three-dimensional volume used in fMRS (red). (**b**) Compared to rest, perceived stimulation upregulated the neurometabolic response in the primary visual cortex, as indicated by increased concentration of lactate and glutamate in this area. This response did not take place during the unperceived condition. (**c**) The neurovascular response was enhanced in the secondary visual cortex (highlighted in darker shades of grey) only for the perceived condition.

Image credit: Brain images in Figure 1a, 1b and 1c are adapted from illustrations by the Database Center for Life Science (CC-BY-SA-2.1-jp); the panel in Figure 1b is adapted from DiNuzzo et al., 2022 (Figure 3—figure supplement 5; CC BY 4.0)

DiNuzzo et al. then used fMRS to measure the changes in glutamate and lactate concentration during perceived and unperceived stimulation compared to rest. They found increased lactate and glutamate in the primary visual cortex for perceived – but not unperceived – stimulation (Figure 1b). Perception may therefore upregulate energy metabolism so that information can be forwarded from the primary to the secondary visual cortex.

In conclusion, combining BOLD-fMRI and fMRS allowed DiNuzzo et al. to show that similar neurovascular responses (as measured by BOLD-fMRI) may reflect different neurometabolic responses (as measured by fMRS) depending on whether a stimulus is perceived. In fact, the results suggest that, compared to neurovascular responses, changes in neurometabolic responses may be a more appropriate indicator of visual perceptual processes and output activity from primary to secondary visual regions. Over the past 20 years, BOLD-fMRI has been one of the most common brain imaging methods for investigating human cognition non-invasively; the study by DiNuzzo et al. now further supports the need to combine approaches in brain imaging to better decipher precise neuronal processes.
